# Interspecific Bacterial Interactions are Reflected in Multispecies Biofilm Spatial Organization

**DOI:** 10.3389/fmicb.2016.01366

**Published:** 2016-08-31

**Authors:** Wenzheng Liu, Henriette L. Røder, Jonas S. Madsen, Thomas Bjarnsholt, Søren J. Sørensen, Mette Burmølle

**Affiliations:** ^1^Molecular Microbial Ecology Group, Section of Microbiology, Department of Biology, University of CopenhagenCopenhagen, Denmark; ^2^Department of Immunology and Microbiology, Faculty of Health Sciences, University of CopenhagenCopenhagen, Denmark; ^3^Department of Clinical Microbiology, Copenhagen University HospitalCopenhagen, Denmark

**Keywords:** multispecies biofilms, interspecies interactions, spatial organization

## Abstract

Interspecies interactions are essential for the persistence and development of any kind of complex community, and microbial biofilms are no exception. Multispecies biofilms are structured and spatially defined communities that have received much attention due to their omnipresence in natural environments. Species residing in these complex bacterial communities usually interact both intra- and interspecifically. Such interactions are considered to not only be fundamental in shaping overall biomass and the spatial distribution of cells residing in multispecies biofilms, but also to result in coordinated regulation of gene expression in the different species present. These communal interactions often lead to emergent properties in biofilms, such as enhanced tolerance against antibiotics, host immune responses, and other stresses, which have been shown to provide benefits to all biofilm members not only the enabling sub-populations. However, the specific molecular mechanisms of cellular processes affecting spatial organization, and vice versa, are poorly understood and very complex to unravel. Therefore, detailed description of the spatial organization of individual bacterial cells in multispecies communities can be an alternative strategy to reveal the nature of interspecies interactions of constituent species. Closing the gap between visual observation and biological processes may become crucial for resolving biofilm related problems, which is of utmost importance to environmental, industrial, and clinical implications. This review briefly presents the state of the art of studying interspecies interactions and spatial organization of multispecies communities, aiming to support theoretical and practical arguments for further advancement of this field.

## Introduction – Spatial Organization in Biofilms

Microorganisms typically live in dense multispecies communities with distinct patterns of spatial organization, termed biofilms. This specific mode of living generally provides strong fitness advantages to biofilm-associated bacteria compared to their planktonic counterparts (Hoiby et al., 2010; Bjarnsholt et al., 2013; Kragh et al., 2016), making biofilms ubiquitously present in most bacterial habitats including the human body (Burmølle et al., 2010; Bjarnsholt, 2013). Having established the general circumstances for biofilm formation in monocultures, the focus in biofilm research has in recent years been gradually moving toward investigating the complexity and interactions in multispecies biofilms (Zengler and Palsson, 2012; Burmølle et al., 2014).

Observations from numerous studies converge on the finding that bacteria residing in multispecies biofilms show spatial positioning in response to interspecific interactions, which is crucial for community functions (Momeni et al., 2013; Burmølle et al., 2014). For instance, the well-studied oral biofilms show complex organized microbial structures due to interspecies interactions (Zijnge et al., 2010; Ferrer and Mira, 2016). In other cases, bacterial species tend to keep proper distance, thereby avoiding strong substrate competition or toxic compounds secreted by others (Kim et al., 2008), illustrating that different types of interactions can lead to distinct types of spatial organization (Satoh et al., 2007; Momeni et al., 2013). In general, spatially structured environments are believed to facilitate a more effective coexistence of bacterial species, due to a negation of localized competitive interactions as well as the stabilization of beneficial interactions such as co-metabolism and coordinated social activities (Hansen et al., 2007; Pande et al., 2015). This in turn has been illustrated to increase virulence during infections (Harrison et al., 2006).

Visual analysis of biofilm communities is one of the most common tools applied in the study of biofilm model systems as well as natural systems, including *in vivo* settings. Therefore, the ability to correlate mechanisms of interspecies interactions to the spatial organization of multispecies biofilms is vital for enhancing our understanding of the function and dynamics in these systems. Recent technological advances have resulted in lowered costs and improved resolution. Methods such as next generation sequencing (Medini et al., 2008), advanced mass spectrometry (Park et al., 2014), and high resolution microscopy (Neu and Lawrence, 2015), enable the generation of ample amounts of data to provide more precise analyses of complex communities. However, how interspecies interactions affect spatial organization, and vice versa, are still poorly understood. Therefore, a better understanding of spatial organization based on relevant studies might provide an alternative approach to reveal interspecies interactions of constituent species in multispecies communities.

The main purpose of this review is to establish that there is a link between interspecies interactions and spatial organization of bacterial species in *in vitro* multispecies biofilms. Species residing in microbial communities with complex structures frequently affect the growth of the others by both cooperative and competitive interactions (Kim et al., 2008; Nadell et al., 2009), and these interplays might change with the development of the community in response to both cell-cell and cell-environment interactions (Coyte et al., 2015). Additionally, the existing methods and the emerging high-resolution techniques, suitable for application in visualizing spatial distribution of bacterial species in complex communities, will be briefly described.

## Biological Processes Affecting the Spatial Organization of Microbes in Multispecies Biofilms

A central tenet of biofilm research is that biofilm formation is a dynamic and cyclic process. Up until now, current models describe biofilm formation as a circular process initiated by free-floating microbial cells attaching to a surface, followed by growth into a mature structurally complex biofilm and culminating in the dispersal of detached cells from the biofilm, allowing colonization of new niches (Tolker-Nielsen et al., 2000; Sauer et al., 2002; Hall-Stoodley et al., 2004; Monds and O’Toole, 2009). However, a new study has challenged this model by showing that aggregates have a fitness advantage when initiating biofilms compared to planktonic bacteria (Kragh et al., 2016). Interspecies interactions, including co-metabolism, quorum sensing and production of antimicrobial compounds have been shown to play important roles in regulating microbial activities *in vitro* (Hojo et al., 2009), and have important functions in shaping the spatial structure of biofilms. It seems that bacterial species organize in three general forms; interspecific segregation, co-aggregation, and/or stratification, based on different types of interspecies interactions. Momeni et al. (2013) have partly verified this by combining computational models and experimental work, indicating that only strong inter-population cooperation leads to partner intermixing in microbial communities. In the following sections, we will discuss the interconnection between different types of interspecies interactions and spatial organization of microbes in multispecies biofilms.

## The Role of Metabolic Interactions

Metabolic interactions, leading to cooperation, exploitation, or competition, are ubiquitous in multispecies biofilm and play important roles in maintaining the diversity and stability of microbial communities (Møller et al., 1998; Embree et al., 2015; Zelezniak et al., 2015).

Based on evolutionary theory, it has been suggested that spatial intermixing favors the evolution of metabolic mutualism because it keeps mutualistic partners in close proximity, thereby allowing for stronger reciprocity, which in turn facilitate the exchange of metabolites between partners (Doebeli and Knowlton, 1998; Foster and Wenseleers, 2006). In fact, this was experimentally demonstrated by Pande et al. (2015), who found that cooperative cross-feeding was much more favored in spatially structured environments, such as biofilms, compared to mixed, non-structured environments. In addition, Palmer et al. (2001) studied the interspecific interactions of three pairwise co-aggregating oral bacterial strains composed of *Streptococcus gordonii*, *Streptococcus oralis*, and *Actinomyces naeslundii*. Of these three species, two (*S. oralis* and *A. naeslundii*) were incapable of growing as mono-species biofilm, however, both of them showed a luxuriant, intermixing growth pattern when co-cultured on a glass surface using saliva as the sole nutrient source, indicating strong metabolic interdependence between them.

Interestingly, in contrast to intermixing patterns observed in cases of strong metabolic interdependence (mutualism), Estrela and Brown (2013) stated that weak metabolic interdependence results in a spatial structure with initial species segregation. Likewise, exploitative and competitive interactions driven by limited nutrients and space during the development of biofilms lead to greater interspecific segregation. In accordance, Christensen et al. (2002) observed this phenomenon by performing a study showing the shift from weak cooperation to exploitation of two species co-cultured in biofilm. This lead to a layered structure with patchy patterning of species as the biomass increased, with the benefitting species overgrowing the one being benefitted from. Hansen et al. (2007) also found a similar pattern formed between two species, as a result of a recent mutation in the overgrowing strain. This novel exploitative interaction could, however, be transient, since it generates a selective pressure on the producing strain to avoid being exploited.

Competitive metabolic interactions among species often play critical roles in shaping the structure and function of multispecies communities. Scheffer and van Nes (2006) demonstrated that self-organized segregation of species in communities is a direct effect of competitive interactions, using a model based on classical competition theory. Kim et al. (2008) and Momeni et al. (2013) further verified this by performing competitive experiments in mixed species communities, indicating that biofilms with competitive pairs frequently show a spatial structure with interspecific segregation (equal-fitness competition) or one species dominant (unequal-fitness competition).

## Chemical Heterogeneity in Multispecies Biofilms

It is well recognized that planktonic, well-mixed conditions result in bacterial cell populations with fairly uniform physiological activity, whereas physiological heterogeneity is common in biofilms due to chemical gradients resulting from the spatial structure (Lee et al., 2005; Gu et al., 2013). Specifically, the extracellular polymeric substances (EPS) provide biofilms a physical structure that segregates microenvironments with different biochemical properties (Lawrence et al., 2007; Madsen et al., 2015), in which bacteria respond and adapt to the local chemical conditions, leading to biological heterogeneity (Xu et al., 1998; Stewart and Franklin, 2008).

As discussed above, metabolic interactions play important roles in the spatial organization of species in multispecies biofilms, which is an indirect reflection of chemical heterogeneity. It has been shown that the layered structure of oral biofilms is a consequence of strong interspecific cooperation among anaerobes and aerobes (Kolenbrander et al., 2006; Mark Welch et al., 2016). Moreover, the chemical heterogeneity is verified in numerous studies using microsensors to detect microscale concentration profiles for a number of compounds in biofilms (Schramm et al., 2000; Chae et al., 2012; Sun et al., 2014), showing that the distribution of these compounds was closely related to that of species in biofilms, which is most likely mechanistically explained by specific metabolic interactions. Therefore, there is potential for elucidating interspecies interactions in detail, by using microelectrodes combined with other techniques, including meta-omics analysis (Embree et al., 2014), fluorescent *in situ* hybridization (FISH; Almstrand et al., 2013) or stable isotope labeling (Verastegui et al., 2014).

## The Connection Between Community Structure and Horizontal Gene Transfer

Biofilm offers an environment with a high cell density, making it well suited for horizontal gene transfer (HGT) (Sørensen et al., 2005). Genes that are transferred horizontally can provide a vast array of new phenotypes, e.g., adjustment of the host metabolic level, resistance toward antibacterial compounds or the ability to form biofilm, thereby facilitating a wide range of adaptions. It has been shown that the presence of conjugative plasmids stimulates biofilm development and modifies the biofilm structure (Ghigo, 2001; Reisner et al., 2006), and the mechanism has been assigned to the plasmid encoded conjugative pili facilitating enhanced attachment and biofilm formation. Reisner et al. (2003) found that cell attachment and microcolony formation were similar for *Escherichia coli* isolates with and without IncF plasmids, however, expansion of the biofilm structure only occurred for conjugative plasmid-carrying strains resulting in a 70–100 μm thick structure. We observed a different effect when co-culturing *Pseudomonas putida*, *E. coli* and *Kluyvera* sp. in multispecies biofilms; when *P. putida* harbored the conjugative plasmid pKJK5, the biofilm-attached biomass decreased (Røder et al., 2013). This was also the case when *P. putida* was grown as single species biofilm with and without the plasmid. In addition, fimbriae encoding genes have been identified on various plasmids and were found to stimulate biofilm formation (Burmølle et al., 2008, 2012).

The studies mentioned above exemplify how biofilm structure can be affected by plasmids; however, biofilm also allows for the possibility of maintaining the plasmids. Biofilms have been proposed to facilitate the maintenance of plasmids over time by allowing plasmids to be retained in the inactive parts of the biofilm, dominated by dormant cells and thus devoid of growth competition (Madsen et al., 2014).

## Spatial Organization Reflects and Drives the Function of Multispecies Communities

The development of multispecies biofilms is believed to proceed as a succession of cooperative and competitive events, which are influenced by cell-cell and cell-environment interactions. The continuously changing cell-cell interactions, resulting from the heterogeneity in biofilms, are considered a main force driving the function of multispecies communities (Stewart and Franklin, 2008).

An established example showing this is dental plaque in which species spatially organize in a stratified structure (Marsh et al., 2011; Mark Welch et al., 2016), which is believed to be largely affected by co-aggregation interactions mediated by functional adhesins located on the cell surface (Kolenbrander et al., 2006). Within the structure, individual taxa are located at micron scales in a non-random way suggestive of their functional niches in these communities (Mark Welch et al., 2016). Co-aggregation has been shown to strongly impact the development of oral multispecies communities, because only specific secondary and late colonizers can be adopted into the already attached biofilm (Rickard et al., 2003), which is believed to be important for the proper establishment and function of these communities. However, the effect of co-aggregation on the development and spatial organization of non-oral multispecies communities is still poorly understood.

Spatial organization has been demonstrated to play an important role in reducing the strength of between-species cooperative interactions, contributing to the stability of multispecies communities, such as gut microbiome (Coyte et al., 2015). It is predicted that the host can benefit from zoning species within gut microbiome in order to maintain community stability and species diversity. This strategy, used by the microbes for development of more stable and diverse communities, has similarities to that utilized by plants and animals in nature, which stabilizes ecosystems by branching the food chain into a web with spatial structure (McCann, 2000). Several model investigations have shown that natural food-web structures can, indeed, enhance ecosystem stability (McCann and Hastings, 1997; Huxel and McCann, 1998; Post et al., 2000). Moreover, the specific patterning of microbes leads to increased biomass and enhanced tolerance toward antibiotics compared to their component species individually (Ahmed et al., 2009; Kolenbrander et al., 2010; Lee et al., 2014). Therefore, a dual focus on both spatial organization and multispecies biofilms succession will help identify entry points to uncover the molecular mechanisms of the processes, in order to address biofilm related problems.

## Methods to Examine and Link the Spatial Organization to Biological Activities

The key to better understand the forces shaping the structure of biofilms is found in linking the visual observations to specific processes. This can be achieved by selecting appropriate biofilm model systems and high-resolution techniques for analysis (Røder et al., 2016).

Confocal laser scanning microscopy (CLSM) has become a standard technique for structural investigation of hydrated microbiological samples at the microscale. Two- or multiphoton microscopy provides new options for *in situ* imaging, such as visualizing thick specimens and excitation of ultraviolet fluorochromes. In order to visualize the bacterial cells in biofilms, different probes or differential staining approaches have been applied to distinguish potential interference of fluorochromes (Muffler and Ulber, 2014). Specifically, fluorescent protein, immunofluorescence techniques (Bao et al., 2014) and FISH (Almstrand et al., 2013) are the most commonly used techniques targeting specific bacterial groups or species. Many advanced versions of the FISH technique have been implemented for different purposes, most commonly; catalyzed reporter deposition-FISH (CARD-FISH) for fluorescent signal enhancement (Lupini et al., 2011), peptide nucleic acid-FISH (PNA-FISH) for better penetration and faster hybridization (Werthén et al., 2010) and CLASI-FISH for expanding the number of distinguishable taxa in complex communities (Valm et al., 2012). In addition, an innovative technique, laser ablation electrospray ionization mass spectrometry (LAESI-MS), has been used for studying the spatial distribution of mixed species biofilms and is showing potential advantages contrasted to CLSM experiments (Dean et al., 2015), as LAESI-MS allows for rapid analysis of unfixed and wet biofilms.

Data processing software, such as Daime (Daims et al., 2006), has been widely used for analyzing co-localization of species in multispecies biofilms (Schillinger et al., 2012; Almstrand et al., 2013), which can be used as an indicator for tracking the shifts of interspecific interactions with the development of the communities. In addition, simple model systems, composed of few species, may be valuable to predict interactions in more complex communities (Ren et al., 2015).

It should be emphasized that the function of a biofilm community cannot be inferred solely from the spatial organization of the sessile organisms without information about their respective metabolites or specific activities. However, recently developed technologies show great promise in providing this additional vital information. As examples, matrix-assisted laser desorption/ionization (MALDI) and laser ablation inductively coupled plasma (LA-ICP) have recently been successfully combined with imaging mass spectrometry (IMS) to visualize proteins and metal distributions within biofilms, demonstrating that metal fluctuations play important roles in microbial community structure (Wakeman et al., 2016). In addition, meta-transcriptomics, in combination with single-cell genome sequencing, has been used to uncover the complicated microbial interactions and metabolic capabilities of an alkane-degrading methanogenic community (Embree et al., 2014). Tracing of isotope labeled substrates can be adapted to reveal the flow of metabolites in complex microbial communities in conjunction with metagenomic analysis, enabling identification of functional genes in different species (Verastegui et al., 2014). Combining such chemical and genetic analysis with detailed information of the spatial organization of multispecies biofilm will facilitate linking the spatial organization to the biological activities.

## Conclusion

Space is an important factor that can either facilitate or hamper interactions between cells. This of course is dependent on the physio-chemical nature of the interaction itself, whether e.g., it is expedited via a diffusible molecule or cell-cell contact. However, due to the vast number of interactions occurring in multispecies biofilms it is, in many cases, much cumbersome to try and fully understand each interaction. Moreover, understanding overall mechanisms of interactions and cellular processes that affect spatial organization, and vice versa, can provide a means of associating spatial distribution of different bacteria and how they interact. Therefore, the better understanding of spatial organization based on relevant studies would be an attractive alternative approach that might reveal interspecies interactions of constituent species in multispecies communities. Closing the gap between visual observation and biological processes may become crucial for resolving biofilm related problems, which is of utmost importance to environmental, industrial, and clinical implications.

**FIGURE 1:**
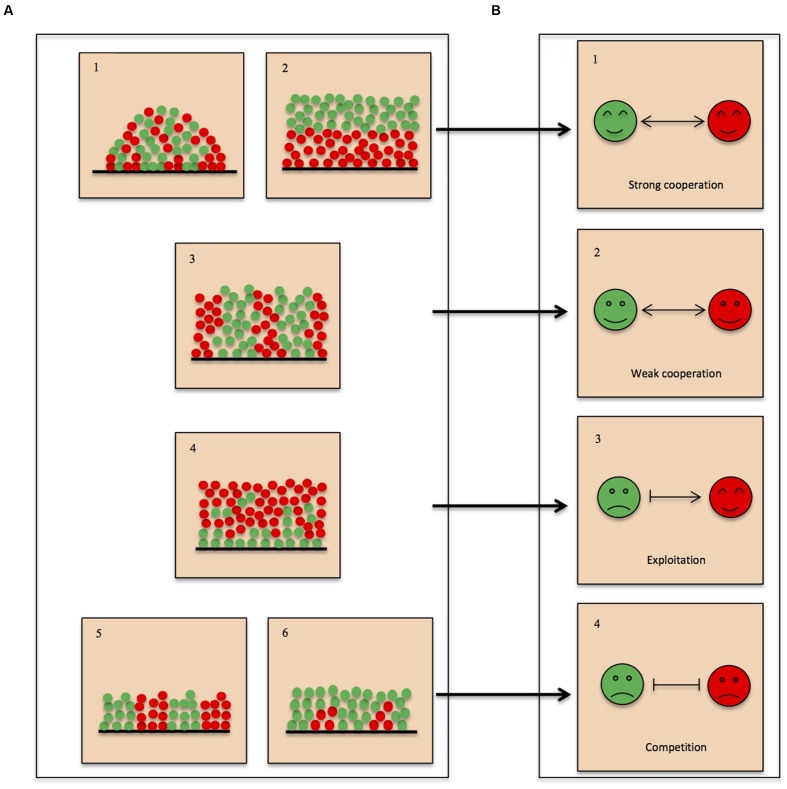
**Linking interspecific interactions to spatial organizations in multispecies biofilms.** **(A)** Spatial organization of microbes in biofilms. Based on observation, there are five general forms in which the bacteria are organized: (1) intermixing; (2) layered structure without patchy patterning; (3,5) interspecific segregation; (4) layered structure with patchy patterning; (6) patchy patterning structure. Both (4) and (6) represent that one species is dominant in the biofilms. **(B)** Interspecific interactions. Interspecific interactions are divided into three groups based on whether species residing in multispecies communities benefit or suffer from the specific interaction: (1,2) cooperation; (3) exploitation; (4) competition. Cooperation and exploitation lead to increased biomass of one or all member species in mixed species compared to single-species biofilms (**A-**1,2,3,4), whereas competition results in decreased biomass of all member species in mixed species compared to single-species biofilms (**A**-5,6). Respectively, arrows and vertical bars represent growth facilitation and inhibition.

The fitness effects of interspecific interactions are believed to be a major driving force of spatial organization of microbes in multispecies biofilms. Simulations derived from an individual-based fitness model showed that deviations caused by indirect interactions can obscure direct interactions and lead to misinterpretations (Momeni et al., 2013). Additionally, interspecific interactions between competitive pairs could shift to indirect cooperation for coexistence in response to the surrounding species or resources (Wootton, 2002). Even so, there is apparent and predictable correlation between interspecific interactions and spatial organization of microbes in multispecies biofilms, as illustrated in **Figure 1**: strong interdependence (**Figure 1B-1**) favors intermixed distribution (**Figure 1A-1**) or layered structure (**Figure 1A-2**), mainly dependent on the metabolic properties of the microbes, whereas weak interdependence (**Figure 1B-2**) is reflected in spatial structures with interspecific segregation when there is no nutrient and space limitation (**Figure 1A-3**); layered structure with patchy patterning (**Figure 1A-4**) implying exploitation (**Figure 1B-3**); mutual inhibition (**Figure 1B-4**) will result in decreased biomass with patchy patterning (unequal-fitness competition; **Figure 1A-5**) or interspecific segregation (equal-fitness competition; **Figure 1A-6**). Hence, detailed description of spatial organization in multispecies biofilm using advanced *in situ* imaging techniques in combination with recently developed technologies can reveal the molecular mechanisms underpinning interspecies interactions and bring biofilm research an important step forward.

## Author Contributions

WL and HR conducted the literature study and wrote the draft manuscript. TB, JM, SS, and MB edited the manuscript.

## Conflict of Interest Statement

The authors declare that the research was conducted in the absence of any commercial or financial relationships that could be construed as a potential conflict of interest.
